# How an assembly factor enhances covalent FAD attachment to the flavoprotein subunit of complex II

**DOI:** 10.1016/j.jbc.2022.102472

**Published:** 2022-09-08

**Authors:** Elena Maklashina, Tina M. Iverson, Gary Cecchini

**Affiliations:** 1 Molecular Biology Division, San Francisco VA Health Care System, San Francisco, California, USA; 2 Department of Biochemistry & Biophysics, University of California, San Francisco, California, USA; 3 Department of Pharmacology, Vanderbilt University, Nashville, Tennessee, USA; 4 Department of Biochemistry, Vanderbilt University, Nashville, Tennessee, USA; 5 Department of Center for Structural Biology, Vanderbilt University, Nashville, Tennessee, USA; 6 Department of Vanderbilt Institute of Chemical Biology, Vanderbilt University, Nashville, Tennessee, USA

**Keywords:** succinate dehydrogenase, fumarate reductase, complex II, covalent flavins, mitochondrial assembly factors, FrdA, free flavoprotein subunit of *E. coli* quinol:fumarate oxidoreductase, SdhA, free flavoprotein subunit of *E. coli* succinate:ubiquinone oxidoreductase, SDHA, free flavoprotein of human complex II, SdhE, *E. coli* covalent flavin assembly factor, SDHAF2, human covalent flavin succinate dehydrogenase assembly factor, TCA, tricarboxylic acid

## Abstract

The membrane-bound complex II family of proteins is composed of enzymes that catalyze succinate and fumarate interconversion coupled with reduction or oxidation of quinones within the membrane domain. The majority of complex II enzymes are protein heterotetramers with the different subunits harboring a variety of redox centers. These redox centers are used to transfer electrons between the site of succinate–fumarate oxidation/reduction and the membrane domain harboring the quinone. A covalently bound FAD cofactor is present in the flavoprotein subunit, and the covalent flavin linkage is absolutely required to enable the enzyme to oxidize succinate. Assembly of the covalent flavin linkage in eukaryotic cells and many bacteria requires additional protein assembly factors. Here, we provide mechanistic details for how the assembly factors work to enhance covalent flavinylation. Both prokaryotic SdhE and mammalian SDHAF2 enhance FAD binding to their respective apoprotein of complex II. These assembly factors also increase the affinity for dicarboxylates to the apoprotein–noncovalent FAD complex and stabilize the preassembly complex. These findings are corroborated by previous investigations of the roles of SdhE in enhancing covalent flavinylation in both bacterial succinate dehydrogenase and fumarate reductase flavoprotein subunits and of SDHAF2 in performing the same function for the human mitochondrial succinate dehydrogenase flavoprotein. In conclusion, we provide further insight into assembly factor involvement in building complex II flavoprotein subunit active site required for succinate oxidation.

Many proteins and enzymes exist in large multisubunit assemblies that are required for the full functionality of the complex. The assembly of such complexes is not spontaneous and is carefully guided by various chaperones and specific assembly factors (1, 2, 3). An important part of this process is correct incorporation of different prosthetic groups into individual proteins before complex assembly begins. Complex II (succinate dehydrogenase, SDHABCD) is one such example of a protein assembly that is useful for the study of protein assembly pathways.

Complex II is membrane bound and plays a central role in mitochondrial metabolism where it is part of the tricarboxylic acid (TCA) cycle oxidizing succinate to fumarate, and it also functions in electron transport and energy production by reducing coenzyme Q_10_ (4, 5). When anaerobic or anoxic conditions lead to high reduction levels of coenzyme Q_10_, complex II also may catalyze the reverse reaction with fumarate serving as an electron acceptor in many types of mammalian tissues (6, 7, 8, 9). Complex II is comprised of two domains; a soluble heterodimer (SDHA–SDHB) catalyzing succinate–fumarate interconversion and a transmembrane domain that forms a quinone reaction site. The two subunits of the soluble domain, the SdhA flavoprotein and the iron–sulfur protein SdhB, are highly conserved among all complex II proteins (4, 5, 10). The catalytic site for dicarboxylate substrates contains a covalently bound FAD prosthetic group linked through an 8α-*N*(3)-histidyl-FAD bond (11, 12) to the SDHA subunit. An important consequence of the covalent bond is that the redox potential of the flavin cofactor is substantially raised (13, 14, 15, 16). For example, the redox potential of free FAD in solution is *E*_*m*_ = −219 mV, whereas in complex II homologs with covalent FAD, the potential is *E*_*m*_ = ∼−50 to −90 mV (17, 18, 19). The rise in redox potential was confirmed by studies demonstrating that the same proteins genetically engineered to harbor a noncovalent flavin reduced the FAD potential by ∼−90 to −150 mV (13, 20, 21). An important consequence of the rise in FAD redox potential induced by the covalent FAD bond is that complex II enzymes can oxidize succinate and interact with a more diverse group of electron acceptors. For example, enzymes evolutionarily related to the SDHA flavoprotein such as soluble bacterial fumarate reductases (22, 23) and *Escherichia coli* L-aspartate oxidases (24) harbor a noncovalent FAD cofactor and as such are only able to reduce fumarate and are not capable of succinate oxidation.

About 10% of all flavoproteins contain covalently attached flavins predominately *via* a covalent linkage to the isoalloxazine ring (16). Formation of the covalent flavin bond occurs autocatalytically in most flavoproteins by quinone-methide chemistry (25, 26). In complex II, flavinylation of SdhA occurs before assembly of the membrane-bound complex, and the flavinylation reaction requires the presence of the substrate (27, 28), and in eukaryotic complex II and many prokaryotic homologs, an additional protein (SDHAF2/Sdh5/SdhE) is required. Recently, X-ray structures showing the reaction intermediates between the *E. coli* complex II flavoproteins SdhA/FrdA and SdhE and human SDHAF2 and SDHA have become available (27, 29, 30). The overall structures of these assembly intermediates are similar and provide structural insight into how the assembly factor (SdhE/SDHAF2) and a substrate dicarboxylate assist with flavinylation. The structures also indicate that the dicarboxylate is absolutely required in order to maintain the architecture of the flavoprotein in the region where the isoalloxazine ring of the flavin resides in order for quinone-methide chemistry to occur (27). Nevertheless, it is reported that in some thermophilic Archaea (28) and mammalian breast cancer cells (31) that SdhE/SDHAF2 are not required for flavinylation. These findings are somewhat surprising since the X-ray structures show that the binding of SdhE/SDHAF2 provide important constraints to the structure of SDHA/SdhA/FrdA. In addition, it is not clear why nature would evolve an assembly factor such as SdhE/SDHAF2 if it is not truly required to form a functional complex II.

In this article, we investigate the reaction of covalent attachment of FAD to *E. coli* SdhA and FrdA and human SDHA; and the role that the SdhE and SDHAF2 assembly factors play in the reaction. We show that in the *E. coli* complex II flavoproteins SdhA/FrdA, and the human SDHA flavoprotein, formation of the covalent flavin linkage critically depends on the presence of a substrate or its analogs; but can be formed in the absence of the assembly factor. The presence of the assembly factor, however, has multiple effects on the flavinylation reaction. The assembly factor significantly enhances the stability of the flavoprotein complex with FAD. It also increases the rate of the flavinylation reaction. Finally, the assembly factor expands the repertoire of the flavinylation-inducing ligands. These findings help explain why such an assembly pathway has evolved and show how the dicarboxylate is the critical component of the flavinylation reaction.

## Results

### *In vitro* flavinylation of SdhA and FrdA

**Figure 1:**
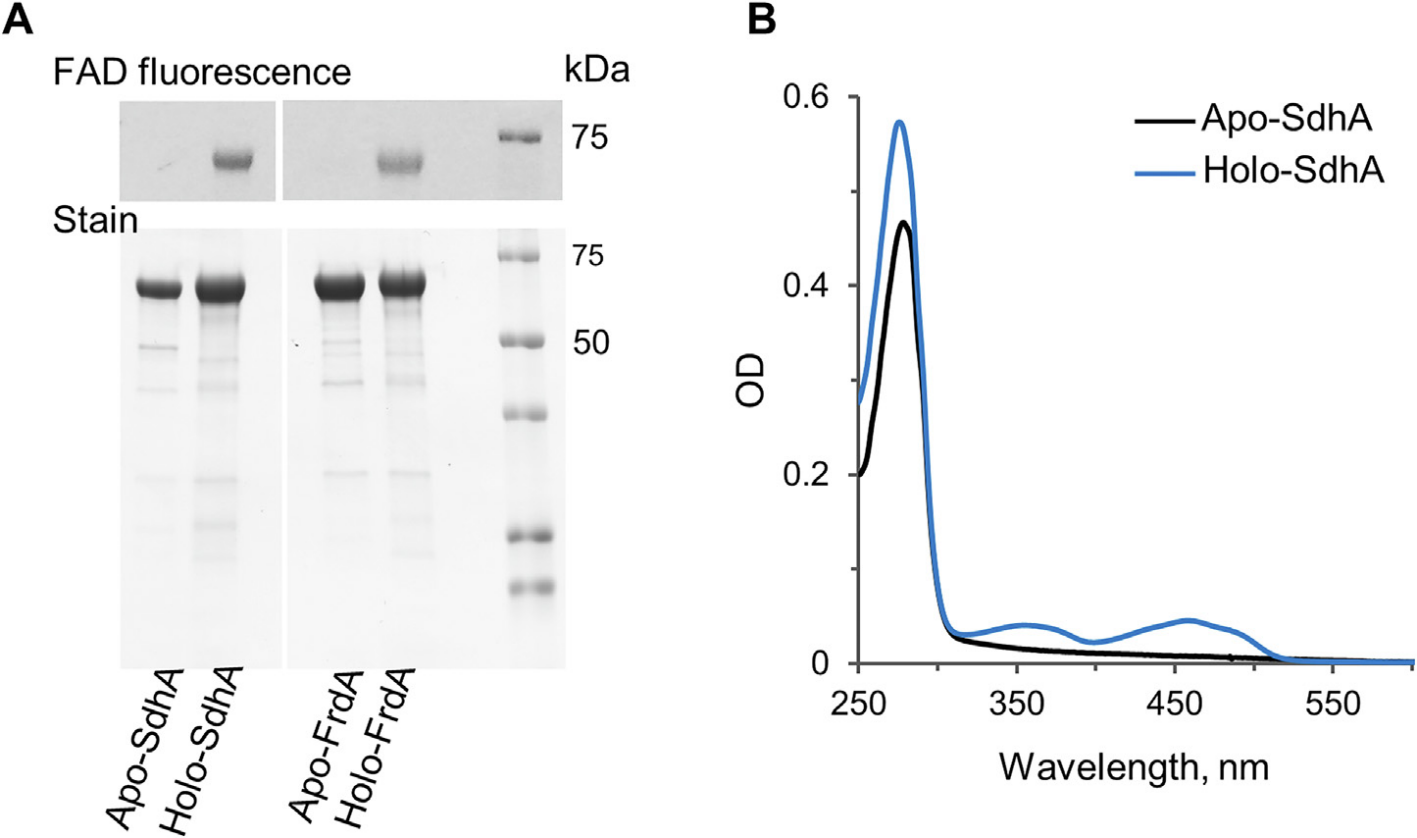
**Isolated apoforms and holoforms of *Escherichia coli* SdhA and FrdA.** Apoproteins were isolated from *E. coli* RP437 (Δ*sdhE*) and holoproteins from *E. coli* BL21(DE3) cells as described (34). *A*, SDS-PAGE of the isolated proteins; UV fluorescence of covalent FAD in SdhA and FrdA is shown in the *top panel*; *bottom panel* shows Coomassie *blue*–stained proteins. *B*, UV–visible spectra of apo-SdhA (*black line*) and holo-SdhA (*blue line*). FrdA, free flavoprotein subunit of *E. coli* quinol:fumarate oxidoreductase; SdhA, free flavoprotein subunit of *E. coli* succinate:ubiquinone oxidoreductase.

The presence of SdhE in *E. coli* has been reported to enhance covalent FAD incorporation into both the complex II homolog flavoproteins, SdhA and FrdA (32, 33). As expected, when SdhA is expressed in Δ*sdhE* background strains under a T5 promoter, the protein lacks covalent FAD (Fig. 1). More surprising is that SdhA isolated from the Δ*sdhE* strain is predominantly in the apoform, that is, lacks noncovalently bound FAD. Indeed, less than 5% of the as-isolated protein contains covalently bound FAD as shown by both fluorescence and spectral analysis (Fig. 1*A* and *B*). The same is true for *E. coli* FrdA, where the isolated apoform is depleted from FAD (Fig. 1*A*).

**Figure 2:**
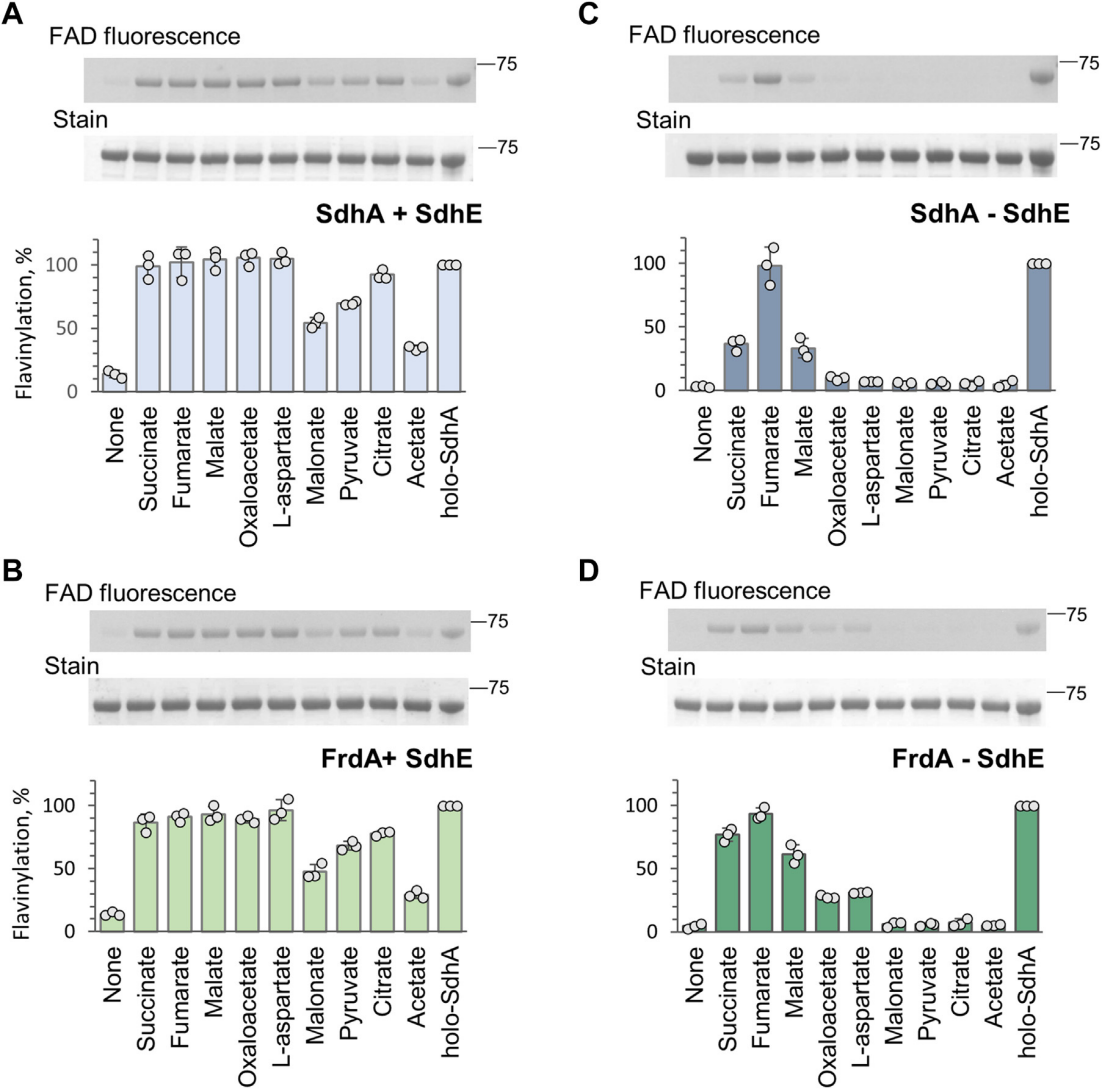
**Covalent flavinylation of apo-SdhA and apo-FrdA.***A* and *C*, covalent FAD attachment in *Escherichia coli apo*-SdhA after incubation with various ligands in the presence (*A*) and absence (*C*) of SdhE. The flavinylation reaction (1 h, 30 ^°^C) was initiated by addition of the indicated ligands (20 mM) to the samples containing apo-SdhA (2.8 μM), FAD (100 μM), and with or without SdhE (10.4 μM). Representative examples of in-gel FAD–SdhA fluorescence and corresponding Coomassie *blue*–stained bands are shown at the *top*. The graphs show quantification of covalently bound FAD. *B* and *D*, the covalent flavinylation of apo-FrdA was performed under the same conditions as in *A* and *C*. The data in (*B*) show flavinylation in the presence or (*D*) the absence of SdhE. Data shown are the mean ± SD (n = 3). FrdA, free flavoprotein subunit of *E. coli* quinol:fumarate oxidoreductase; SdhA, free flavoprotein subunit of *E. coli* succinate:ubiquinone oxidoreductase.

Previous studies of *in vitro* flavinylation of human SDHA and thermophilic SdhA show that the covalent flavinylation reaction was critically dependent upon the presence of dicarboxylates (27, 28). We therefore investigated how complex II substrate analogs stimulate flavinylation in apo-SdhA and apo-FrdA. Both *E. coli* SdhA/FrdA flavoproteins were chosen for analysis because of the available structures of the SdhA–SdhE and FrdA–SdhE assembly complexes (29, 30) and the fact that they are normally found in cells adapted to either aerobic (SdhA) or anaerobic (FrdA) environments. Incubation of apo-SdhA or apo-FrdA in the presence of 100 μM FAD and SdhE for 1 h at 30 ^°^C does not promote covalent flavin attachment (Fig. 2, *A* and *B*). Addition of 20 mM of succinate or fumarate, or their TCA cycle analogs, however, does promote robust flavinylation for either SdhA or FrdA. The four carbon ligands (succinate, fumarate, malate, oxaloacetate, and l-aspartate) show similar responses in stimulating high levels of SdhE-dependent covalent FAD attachment to both SdhA (Fig. 2*A*) and FrdA (Fig. 2*B*). Pyruvate and citrate were also able to induce SdhE-dependent covalent flavinylation as did the three carbon dicarboxylate malonate and two carbon -carboxylate acetate albeit to a lesser extent (∼30%) (Fig. 2, *A* and *B*).

Although the presence of SdhE or its homologs is important for flavinylation of complex II flavoproteins during aerobic growth, we previously reported a significant presence of holo-SdhA/holo-FrdA in cells grown under anaerobic or microaerophilic conditions (34). This suggests that the presence of SdhE or its eukaryotic homologs SDHAF2/Sdh5 may be less important during certain metabolic states such as alteration in TCA cycle metabolites (35) that may occur upon the assembly factor deletion. Therefore, we evaluated the ability of the same set of ligands to promote flavinylation under the same experimental conditions in the absence of SdhE. As shown in (Fig. 2*C* and *D*), fumarate promotes complete SdhE-independent flavinylation of both SdhA (Fig. 2*C*) and FrdA (Fig. 2*D*), whereas succinate and malate are also partially effective. Interestingly, FrdA (Fig. 2*D*) was more amenable to SdhE-independent flavinylation than was SdhA since succinate and malate also induced between 60 and 80% of the flavinylation capacity and even oxaloacetate and l-aspartate were partially effective.

### Comparison of SdhE-dependent and SdhE-independent flavinylation reactions in SdhA

**Figure 3:**
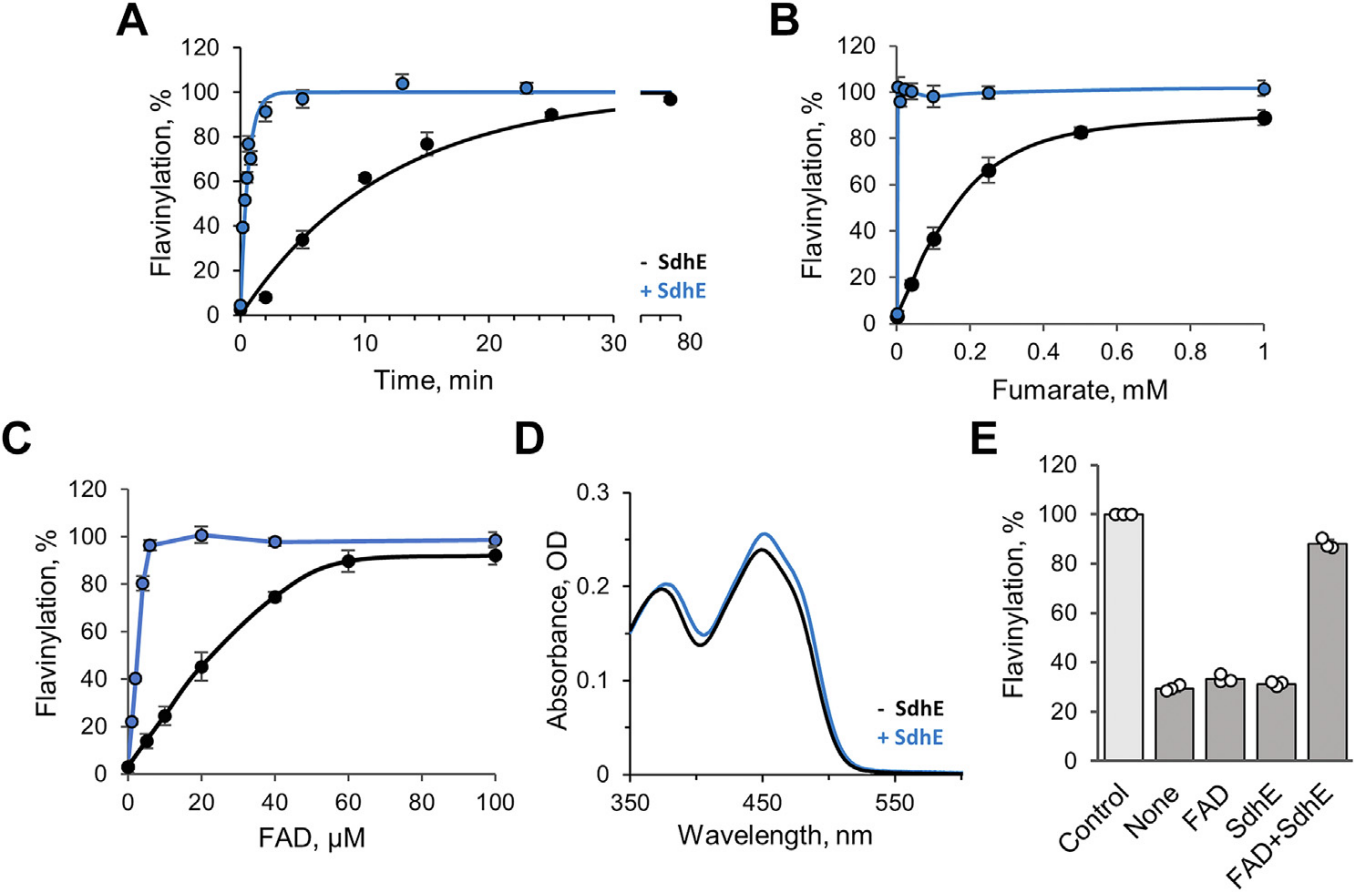
**SdhE enhances flavinylation of SdhA.** Effect of SdhE on (*A*) the time course, (*B*) fumarate dependence, and (*C*) FAD dependence for the flavinylation reaction of apo-SdhA. In all cases, the *blue line* indicates that SdhE was added, and the *black line* represents data in the absence of SdhE. The flavinylation reaction (1 h, 30 ^°^C) was initiated by addition of fumarate. The concentrations of apo-SdhA (6 μM) and SdhE (10.4 μM, when present) were the same in *A*–*C*. Unless indicated, the concentration of fumarate was 10 mM and FAD 100 μM. Data shown are the mean ± SD (n = 3). *D*, SdhE affects the spectral properties of the apo-SdhA–FAD complex. Addition of apo-SdhA (48 μM) to FAD (28 μM) did not elicit noticeable changes in the spectrum of free FAD (*black line*). SdhE (70 μM) was added, and after 5 min, the spectrum was recorded (*blue line*). *E*, SdhE in the presence of FAD protects apo-SdhA during incubation at 37 ^°^C. Apo-SdhA (2.8 μM) was first incubated at 37 ^°^C for 30 min in the presence of FAD (100 μM), SdhE (10.4 μM), or both. The flavinylation reaction was then performed (30 min at 30 ^°^C), and the data are depicted in the graph. Data shown are the mean ± SD (n = 3). SdhA, free flavoprotein subunit of *E. coli* succinate:ubiquinone oxidoreductase; SdhE, *E. coli* covalent flavin assembly factor.

Flavinylation reactions of SdhA and FrdA show a remarkable similarity in response to the stimulating substrate analogs, with fumarate being the only ligand that induced complete flavinylation of the apoproteins regardless of the presence of SdhE. Therefore, we compared the properties of the fumarate-induced flavinylation of apo-SdhA in the presence and the absence of SdhE. First, we examined how SdhE affects the rate of the flavinylation reaction. Figure 3*A* demonstrates the time course of the reaction induced by fumarate. The apparent first-order rate constants (Table 1) are 0.095 min^−1^ for SdhE-independent flavinylation and 1.7 min^−1^ for the SdhE-dependent reaction. Thus, SdhE accelerates flavinylation by nearly 20-fold.

**Table 1:** Effect of dicarboxylate ligands on the covalent flavinylation of *E. coli* apo-SdhA

Ligand	+SdhE		−SdhE	
	*K*^a^, min^−1^	*k*^b^, μM	*k*^a^, min^−1^	*k*^b^, μM
Fumarate	1.7	<1	0.1	120
Succinate	0.7	1		
Malate	0.3	10		
Aspartate	0.1	26		

The reactions were conducted in 50 mM potassium phosphate (pH 7.0) in the presence of 0.1 mM FAD at 30 ^°^C.

Standard error is <10% of the reported values.

a The apparent first-order rate constant.

b Concentration for half-maximal flavinylation after 1 h incubation.

### SdhE modulates affinity to fumarate

The difference between the SdhE-dependent and SdhE-independent flavinylation reactions also extends to the requirement for fumarate, which initiates the reaction. As seen in Figure 3*B*, fumarate by itself induces the half-maximal flavinylation of apo-SdhA at ∼160 μM. In the presence of SdhE, the complete flavinylation of apo-SdhA (6 μM) is achieved with only ∼1 μM fumarate. This suggests that SdhE dramatically increases the affinity between apo-SdhA containing noncovalent FAD and fumarate. For comparison, the *K*_*d*_^fum^ for holo-SdhA–SdhE was found to be 64 μM (29). Thus, fumarate binds significantly tighter to SdhA with noncovalent FAD than it does to holo-SdhA with covalent FAD.

### FAD requirement

We next compared the effect of SdhE on the FAD requirement for the fumarate-induced flavinylation reactions of apo-SdhA. Considering the rate difference for the SdhE-dependent and SdhE-independent flavinylation reactions (Fig. 3*A*), the studies were carried out for 1 h with varied concentration of FAD. The observed half-maximal flavinylation of SdhA was achieved at ∼20 μM FAD when the reaction occurred without added SdhE (Fig. 3*C*). By comparison, in the presence of SdhE, maximum flavinylation was observed at stoichiometric ratios of FAD to apo-SdhA (6 μM), indicating that SdhE significantly improves incorporation of the flavin into apo-SdhA. This enhancement of flavinylation may be due to kinetic differences in the reaction as the presence of SdhE results in a ∼20-fold faster rate for flavinylation compared with that in the presence of apo-SdhA alone (Fig. 3*A*).

In addition to the kinetic differences, SdhE may improve the noncovalent binding of FAD to apo-SdhA and/or the stability of the apo-SdhA–noncovalent FAD complex. Flavin binding to an apoflavoprotein often attenuates the spectral properties of the flavin, and analysis of such optical changes may provide insight into the flavin–protein interaction. Therefore, we examined the spectral changes of FAD in the presence of apo-SdhA and SdhE. Addition of free FAD (28 μM) to apo-SdhA (48 μM) did not elicit noticeable differences in the spectrum; however, following addition of SdhE (70 μM), a noticeable red shift in both the 374 and 450 nm FAD absorbances is observed with an increased absorbance of the 454 nm peak (Fig. 3*D*). This spectral transformation takes several minutes and likely reflects optimization of FAD binding to SdhA because of the stabilizing interactions with SdhE. Although X-ray crystal structures of the FrdA–SdhE and SdhA–SdhE postflavinylation complexes are available (29, 30), there are no corresponding structures of the apoflavoproteins for direct comparison. This precludes a detailed analysis of the structural changes that occur near the flavin upon SdhE binding prior to formation of the covalent flavin bond. Nevertheless, we next evaluated if SdhE influences the binding of FAD to apo-SdhA by estimating the apparent *K*_*d*_^app^ for FAD by the ultrafiltration method previously used to evaluate FAD binding to l-aspartate oxidase (36), the soluble homolog of complex II flavoproteins with noncovalent flavin. Indeed, the estimated *K*_*d*_ for FAD is an order of magnitude lower for apo-SdhA in the presence of SdhE (1.3 ± 0.5 μM) compared with apo-SdhA alone (14.4 ± 1.7 μM); suggesting SdhE does influence the binding of FAD.

### SdhE stabilizes preflavinylation complex of apo-SdhA with FAD

One of the basic functions of a chaperone is to protect its relatively unfolded client protein during a specific stage of maturation (37), and we addressed if SdhE has chaperone-like properties. Apo-SdhA is stable and capable of flavinylation when kept at neutral pH and 4 ^°^C for over 24 h; however, a 30 min incubation at 37 ^°^C diminishes its capacity for flavinylation (Fig. 3*E*). Thus, we investigated if FAD and/or SdhE confers protection for apo-SdhA. The autocatalytic flavinylation of SdhA requires the formation of the SdhA–noncovalent FAD complex in which the apo-SdhA protein folds to accommodate the flavin and also forms the active site where dicarboxylates bind. Nevertheless, as shown in Figure 3*E*, incubation of apo-SdhA with FAD alone does not afford enhanced stability and protection against thermal inactivation of the flavinylation reaction. The same lack of protection for apo-SdhA is seen when only SdhE is present (Fig. 3*E*). It is only when FAD and SdhE are added together to apo-SdhA that protection is elicited.

### Formation of a reduced flavin intermediate upon covalent bond formation

**Figure 4:**
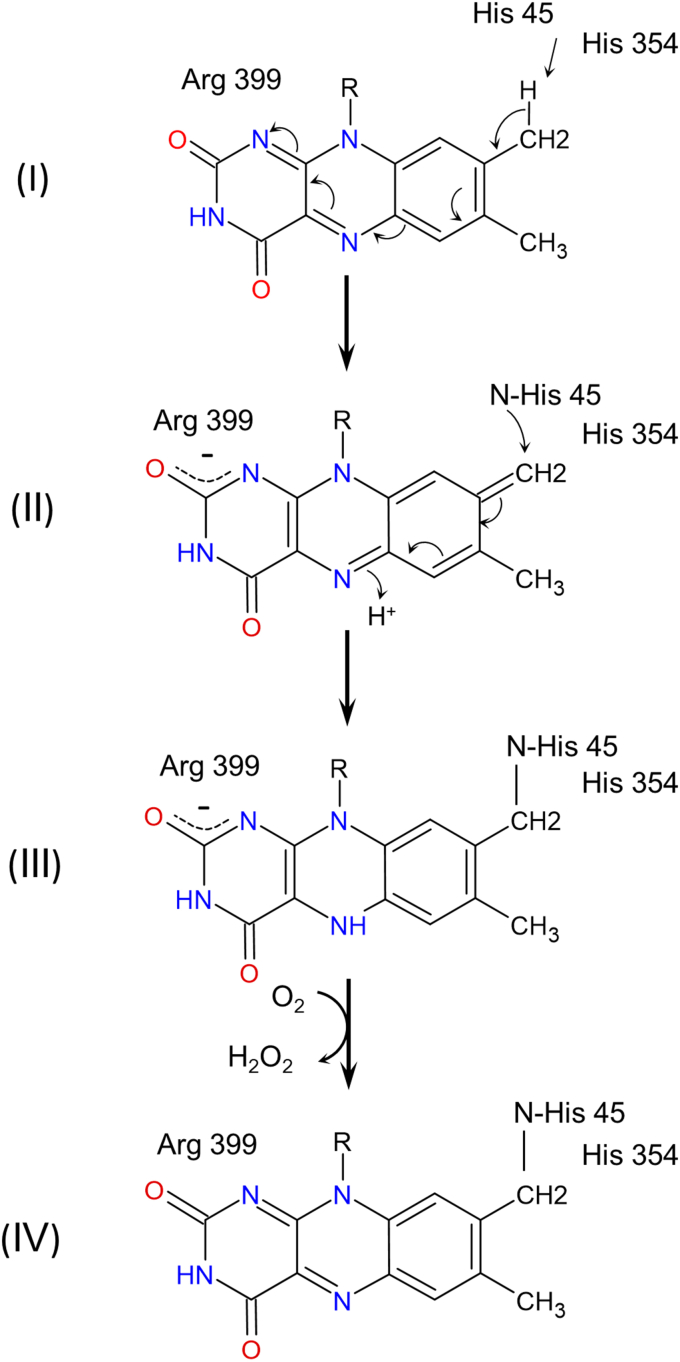
**The quinone-methide mechanism for autocatalytic covalent flavinylation in complex II flavoproteins.** The amino acid numbering is shown for *Escherichia coli* SdhA. See details in the text. SdhA, free flavoprotein subunit of *E. coli* succinate:ubiquinone oxidoreductase.

**Figure 5:**
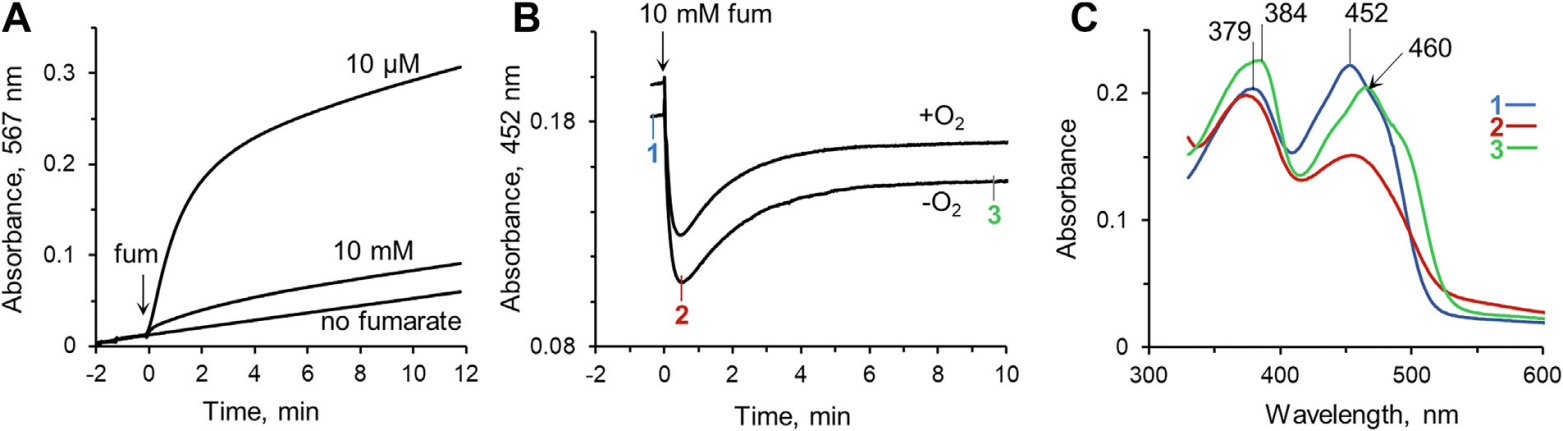
**Reduction/oxidation of FAD during the SdhE-dependent flavinylation reaction.***A*, effect of high (10 mM) and low (10 μM) fumarate on formation of hydrogen peroxide upon SdhE-dependent flavinylation of apo-SdhA is monitored in the presence of Amplex UltraRed. The reaction was performed with apo-SdhA (2.8 μM) in the presence of FAD (2.5 μM), SdhE (4.8 μM), 20 μM Amplex UltraRed, and horseradish peroxidase. *B*, the reduction state of FAD during the flavinylation reaction was monitored in the presence and absence of O_2_ at 30 ^°^C. The reaction was initiated by addition of 10 mM fumarate to a cuvette containing 25 μM apo-SdhA, 20 μM FAD, and 40 μM SdhE. Anaerobic conditions were achieved in the presence of a glucose/glucose oxidase system. Numbers next the curve indicate time points at which spectra from the anaerobic reaction are taken and combined in (*C*). Note the spectrum represented by line 3 is characteristic for the spectrum of oxidized holo-SdhA in the presence of fumarate (29). SdhA, free flavoprotein subunit of *E. coli* succinate:ubiquinone oxidoreductase; SdhE, *E. coli* covalent flavin assembly factor.

Figure 4 demonstrates the postulated quinone-methide mechanism for covalent bond formation (15, 16) using *E. coli* SdhA as the example. As shown in the figure, the flavinylation reaction is initiated by base-assisted deprotonation of the isoalloxazine C(8)-methyl group by one of the nearby His residues (I). The proton abstraction yields an imino-quinone methide intermediate (II) stabilized by a nearby positive charged residue SdhA^R399^. Next, the imidazole ring of the ligating His residue SdhA^H45^ acts as a base to form a covalent bond to the C(8) carbon atom, which results in reduced covalent FAD (III). Under aerobic conditions, the reduced flavin can be reoxidized by oxygen (IV) with formation of hydrogen peroxide as documented for several other covalent flavoproteins (16, 38). Therefore, we tested if hydrogen peroxide is produced during the flavinylation reaction of SdhA. Hydrogen peroxide formation was monitored by the oxidation of Amplex UltraRed (Invitrogen) in the presence of horseradish peroxidase by an increase of absorbance at 567 nm. Figure 5*A* shows the time dependence of fumarate-induced hydrogen peroxide formation for apo-SdhA in the presence of FAD and SdhE. Rather unexpectedly, induction of flavinylation with 10 mM fumarate resulted in a very low release of hydrogen peroxide in comparison with the background reaction observed without the addition of fumarate. A significant increase in hydrogen peroxide, however, was observed when only 10 μM fumarate was added to start the reaction (Fig. 5*A*). Since robust flavinylation is induced in the presence of SdhE by either 10 μM or 10 mM fumarate as judged by covalent FAD incorporation (Fig. 3*B*), the data in Figure 5*A* indicate that the flavin is indeed reduced upon flavinylation, but fumarate prevents oxygen from interacting with the reduced flavin. Similarly, high fumarate or succinate concentrations inhibit hydrogen peroxide production in mature complex II (39, 40). Thus, the newly formed covalent flavin either remained reduced or fumarate, as an electron acceptor, can compete with oxygen for flavin reoxidation. To further investigate this, we directly monitored the reduction state of FAD by following absorbance changes at 452 nm after the flavinylation reaction was initiated with 10 mM fumarate (Fig. 5*B*). Addition of fumarate to a cuvette containing apo-SdhA, FAD, and SdhE induces a biphasic reaction. First, a fast decrease in absorbance (*k*^*app*^ = ∼7 min^−1^) corresponding to reduction of FAD is observed reaching an optical minimum at about 30 s. The second slower phase (*k*^*app*^ = ∼0.1 min^−1^) corresponds to reoxidation of reduced flavin and reaches its equilibrium at about 5 min (Fig. 5*B*). As shown in the figure, both reductive and oxidative phases were the same when the reaction was performed in the presence or the absence of oxygen, indicating that under these conditions, fumarate prevents oxygen from interacting with the flavin and is the preferred electron acceptor. To confirm flavin redox changes during the flavinylation reaction, we compared the spectral profiles of the anaerobic reactions taken at different time points (Fig. 5*B*). These spectra are shown in Figure 5*C*. The spectra demonstrate that data taken at the point with the lowest absorbance from Figure 5*B* (spectrum 2) correspond to the partially reduced flavin, which returns to the oxidized state at the end of the reaction (spectrum 3). Note, spectrum 3 reflects changes in the FAD absorbance from bound fumarate, that is, a characteristic shift of a maximum at 457 nm and increased extinction and shift of a maximum at 384 nm (27, 29). The observed partial flavin reduction reflects the brief equilibrium between the flavinylation reaction and the reoxidation of the reduced holoflavoprotein.

Collectively, these data confirm that the flavinylation reaction proceeds according to the quinone-methide mechanism (15, 16) (Fig. 4) producing the reduced holoflavoprotein that can be oxidized by fumarate or oxygen. The oxidation can occur *via* two possible mechanisms that would be based on the affinity of fumarate to the preflavinylation SdhA–noncovalent FAD complex and to the reduced holo-SdhA (*i.e.*, covalently flavinylated SdhA protein). If the *K*_*d*_ for fumarate for these two complexes is similar and very low (<1 μM, Fig. 3*B*), then the bound fumarate molecule could initiate the flavinylation reaction and oxidize reduced holo-SdhA (thus preventing oxygen access to the flavin). Alternatively, if the *K*_*d*_ for fumarate for the reduced holoflavoprotein is higher than for SdhA–noncovalent FAD, then the flavinylation inducing fumarate molecule would readily dissociate from reduced holo-SdhA allowing flavin oxidation by bulk fumarate or by oxygen. The data from Figure 5 are consistent with the second mechanism and supported by the higher *K*_*d*_ of 64 μM for fumarate determined for the holo-SdhA–SdhE complex (29). Since SdhE-dependent flavinylation can be promoted by a variety of ligands, oxidation of the reduced holoflavoprotein, if needed for further biogenesis steps, likely proceeds *via* oxygen under aerobic conditions.

### Comparison of different ligands for SdhE-dependent flavinylation

One of the advantages that SdhE provided for the flavinylation reaction is the increased repertoire of substrate analogs that promote flavinylation. We thus compared whether other dicarboxylate ligands that provide high levels of covalent FAD incorporation into SdhA (Fig. 2*A*) are equally efficient in promoting this reaction. As shown in Table 1, succinate was reasonably proficient in the covalent flavinylation reaction, but malate and aspartate were less so, as the rate of the reaction significantly decreased, and the required ligand concentration increased. Nevertheless, the data are consistent with the idea that all the tested ligands may function to induce covalent flavinylation *in vivo* depending upon the metabolic conditions of the cell.

### SDHAF2-independent flavinylation of human SDHA

**Figure 6:**
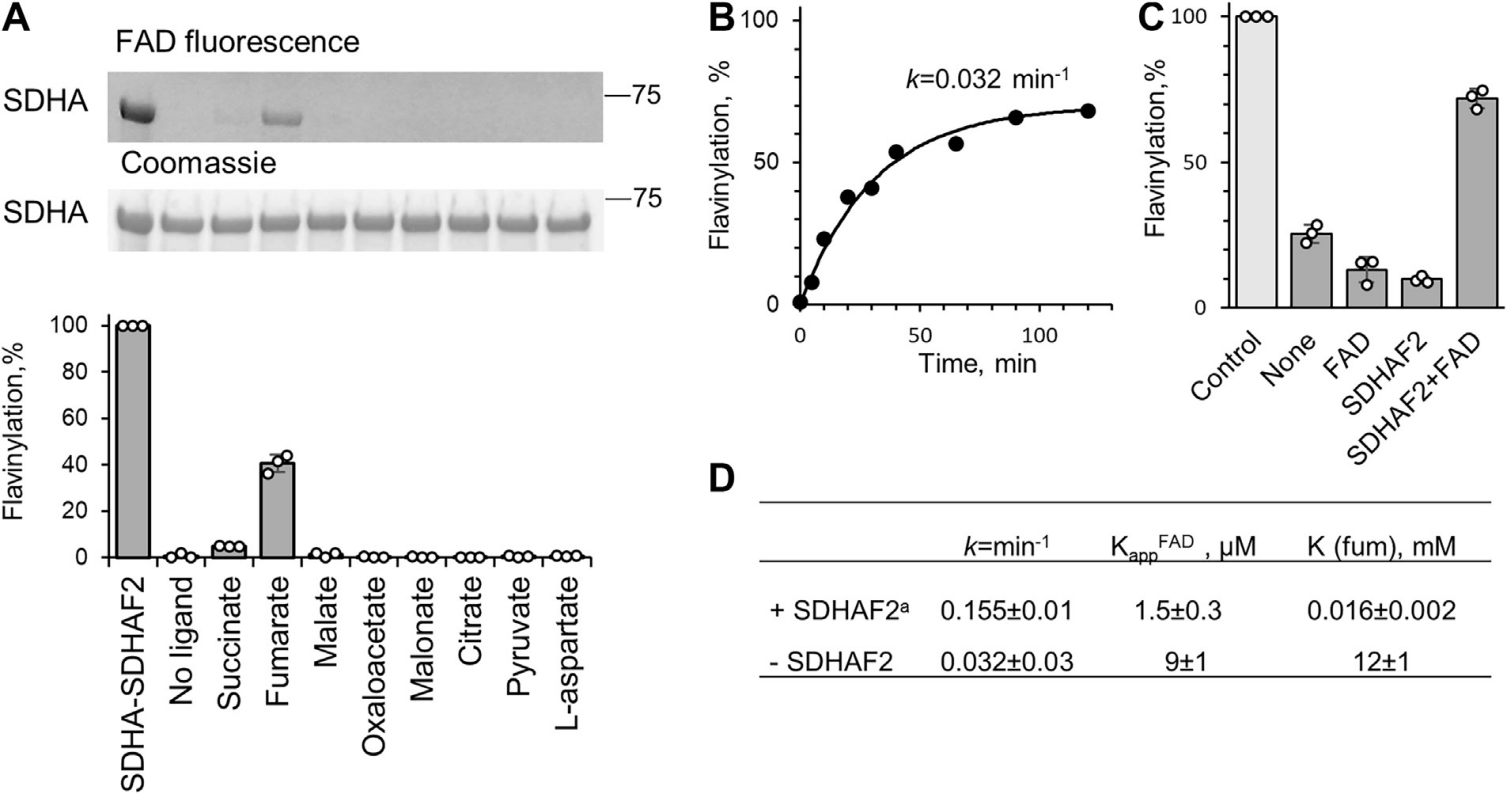
**SDHAF2-independent flavinylation of human SDHA.***A*, *in vitro* flavinylation of human apo-SDHA (3 μM) in the presence of FAD (100 μM) was initiated by indicated ligands (20 mM) and carried out for 1 h at 37 ^°^C. In-gel FAD fluorescence and corresponding Coomassie *blue*–stained SdhA bands are shown (*top panels*, respectively) and quantification of holo-SDHA (*bottom panel*). The isolated holo-SDHA–SDHAF2 complex was used as 100% control. Data shown are the mean ± SD (n = 3). *B*, time course of the fumarate (60 mM) induced SDHAF2-independent flavinylation reaction of SDHA. The rate constant for the first-order reaction was calculated by assuming that 70% of the population of protein was capable for flavinylation. *C*, SDHAF2 in the presence of FAD protects apo-SDHA during incubation (15 min, 37 ^°^C). *D*, comparison of kinetic parameters of apo-SDHA flavinylation reaction with/without SDHAF2. ^a^For comparison, the parameters for the SDHAF2-dependent flavinylation reaction were taken from Ref. (27). SDHAF2, human covalent flavin succinate dehydrogenase assembly factor.

We previously described the SDHAF2-assisted flavinylation reaction of human apo-SDHA (27) and wished to determine if the mammalian system behaved like the bacterial counterparts. The SDHAF2-assisted flavinylation reaction in human SDHA was stimulated by several substrate analogs; however, in comparison to bacterial SdhA, the rate of the covalent flavinylation reaction of SDHA was 0.15 min^−1^ or 10-fold slower than for the bacterial protein (Fig. 3*A*). SDHAF2 is essential for flavinylation and complex II assembly *in vivo* (41), but intriguingly, one study in a human breast cancer cell line showed that KO of SDHAF2 still resulted in formation of complex II containing covalent FAD (31). Thus, whether human SDHA can form a covalent bond to FAD in the absence of SDHAF2 is unclear. Therefore, we investigated whether apo-SDHA could be covalently flavinylated in an SDHAF2-independent manner by dicarboxylates alone in our *in vitro* system, similar to what we observed for *E. coli* SdhA and FrdA (Fig. 2, *C* and *D*). As shown in Figure 6, human SDHA can indeed be covalently flavinylated in an SDHAF2-independent manner, although this reaction requires fumarate and cannot be supported by other dicarboxylates (Fig. 6*A*). Fumarate (20 mM) induced the flavinylation to levels ∼40% compared with that of the flavinylation achieved in the presence of SDHAF2. More detailed analysis of the time course of SDHA flavinylation determined in the presence of 100 μM FAD and 60 mM fumarate (Fig. 6*B*) indicates that this reaction reaches saturation at ∼70% of the level of SDHAF2-dependent flavinylation. This incomplete flavinylation may suggest instability of the isolated apo-SDHA under the given experimental conditions. Indeed, the experimental data were fitted to a single exponential reaction (*k* = 0.032 min^−1^) with the assumption that only 70% of the protein would become flavinylated (Fig. 6*B*). This rate is about fivefold slower than the rate of corresponding reaction determined in the presence of SDHAF2 (27). Consistent with our finding for bacterial SdhA, fumarate-only SDHA flavinylation demonstrates an apparent fivefold increased FAD concentration required to achieve 50% flavinylation in comparison with the SDHAF2-dependent reaction (Fig. 6*D*). Even more dramatic, the concentration of fumarate needed to achieve 50% flavinylation increased from 16 μM to 12 mM if SDHAF2 is not present (Fig. 6*D*). Also similar to what is seen with the bacterial proteins (Fig. 3*E*), the presence of both FAD and SDHAF2 protects human SDHA from thermal inactivation as evidenced by the ability of SDHA to be flavinylated following incubation at 37 ^°^C (Fig. 6*C*). This is consistent with the bacterial and mammalian assembly factors having the same function during the complex II flavoprotein maturation.

## Discussion

Both the flavoprotein and iron–sulfur protein subunits of complex II undergo independent maturation using assembly machinery found in mitochondria or bacterial cells (42, 43, 44, 45). Incorporation of the iron–sulfur centers into the SdhB/SDHB subunit employs the general iron–sulfur cluster machinery (46) and in the case of mitochondrial complex II specific assembly factors such as SDHAF1 and SDHAF3 (44, 47). In some Archaea (28), covalent flavin incorporation in complex II is apparently an autocatalytic reaction; however, many bacteria and eukaryotes require a specific assembly factor. This factor is termed SdhE in *E. coli*, SDHAF2 in mammals, or Sdh5 in yeast (32, 41). In this work, we present data demonstrating that even in organisms that have evolved assembly factors to assist with the covalent attachment of FAD in complex II, the flavoprotein subunits are still capable of forming a covalent flavin bond in a self-catalytic and assembly factor–independent manner. Our data show what advantage the assembly factor provides. For example, several parameters involved in maturation of the flavoprotein are improved: (i) there is an increase in the rate of flavinylation; (ii) an increase in affinity for both FAD and fumarate; (iii) an increase in the number and types of dicarboxylate ligands that can initiate the quinone-methide reaction; and (iv) stabilization of the complex of SDHA with noncovalent FAD.

Three X-ray crystal structures of complex II flavoproteins with their associated assembly factors have become available in recent years (27, 29, 30). These are human SDHA–SDHAF2 with oxaloacetate bound (27), *E. coli* FrdA–SdhE with malonate bound (29), and *E. coli* SdhA–SdhE without a dicarboxylate bound (30). All three structures show that the assembly factor–binding position overlaps with the iron–sulfur protein SdhB/SDHB in mature complex II. It has been proposed that the assembly factor provides the optimal conformation enabling covalent flavin attachment (43). What was not clear, however, was how the assembly factor interacts with the flavoprotein to assist with covalent flavinylation and whether the timing preceded bonding of noncovalent FAD. One possibility is that the assembly factor binds to the apoprotein first shielding it from solvent, stabilizing the folded protein that promotes incorporation of the FAD. Alternatively, the assembly factor binds to the flavoprotein–FAD complex providing the optimal conformation for flavinylation. The data in this work support the latter contention and demonstrate that bacterial and mitochondrial apoflavoproteins form a complex with FAD prior to the interaction with the assembly factor. In the presence of fumarate, the covalent flavinylation can be achieved without the need of the assembly factor. This indicates that acquisition of an assembly factor like SdhE or SDHAF2 is an evolutionary improvement of the autocatalytic flavinylation reaction.

**Figure 7:**
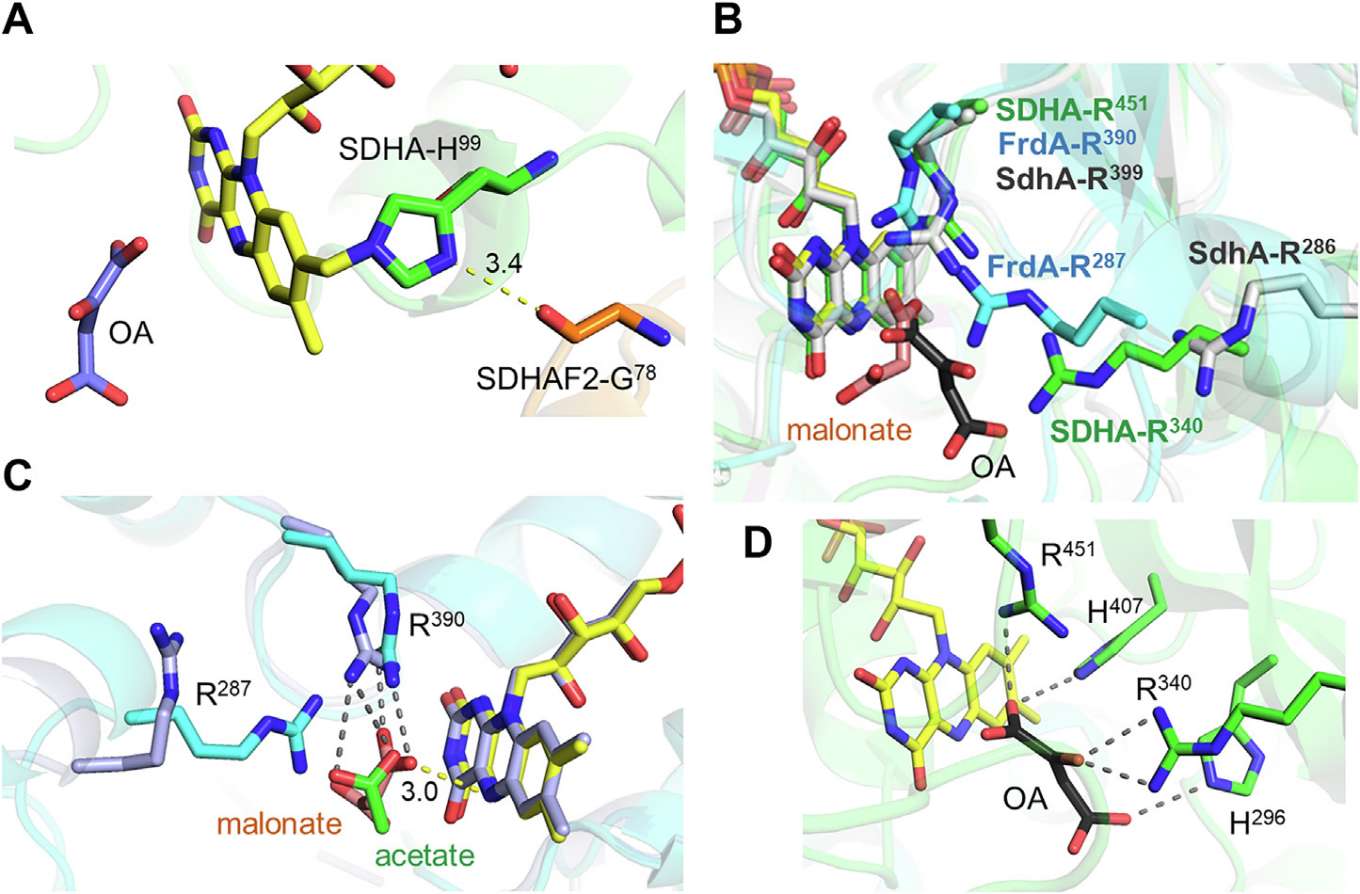
**Flavin environment in complex II flavinylation assembly intermediates.***A*, the hydrogen bond between SDHAF2^G78^ and SDHA^H99^ in human assembly complex SDHA–SDHAF2 (Protein Data Bank [PDB] code: 6VAX). *B*, alignment of three structures of the flavinylation assemblies: SDHA–SDHAF2 (PDB code: 6VAX) shown in *green* with bound oxaloacetate (OA, *black*); FrdA–SdhE (PDB code: 6B58) shown in *teal* with bound malonate (*pink*); SdhA–SdhE with no bound ligand (PDB code: 6C12) shown in *gray*. *C*, comparison of the two nonidentical monomeric FrdA–SdhE complexes from the asymmetric unit found in the crystals. The monomer A with bound malonate (*pink*) is shown in *teal*, and monomer B with bound acetate (*green*) is shown in *light blue*. *D*, polar contacts to OA in the SDHA–SDHAF2 structure. In all panels, polar contacts are shown as *gray dashed lines*, and distances between atoms are shown as *yellow dashed lines*. FrdA, free flavoprotein subunit of *E. coli* quinol:fumarate oxidoreductase; SdhA, free flavoprotein subunit of *E. coli* succinate:ubiquinone oxidoreductase; SdhE, *E. coli* covalent flavin assembly factor; SDHA, free flavoprotein of human complex II; SDHAF2, human covalent flavin succinate dehydrogenase assembly factor.

To form a covalent bond between a nucleophilic amino acid side chain, such as the histidine found in complex II and the C(8)-methyl group of the flavin isoalloxazine ring, a specific conformation of the protein and the flavin must exist. In all three structures of the assembly intermediates (27, 29, 30), a specific interaction between the assembly factor and the flavoprotein has been identified. In the case of human SDHA–SDHAF2 (Fig. 7*A*), the SDHAF2^G78^ carbonyl is positioned within hydrogen bonding distance of the N1 atom of SDHA^H99^, and it was proposed that this interaction helps to orient and activate the flavin-ligating histidine (27, 29). Similar interaction between SdhE^G17^ and the flavin-ligating SdhA^H45^ and FrdA^H44^ residues is present in the structures of the bacterial assembly intermediates (29, 30). The direct action of SDHAF2^G78^ and *E. coli* SdhE^G17^ likely contributes to the 5-fold to 20-fold increased rate of covalent flavinylation observed in the presence of the assembly factor (Figs. 3 and 6) shown in this study.

### Why is a dicarboxylate ligand needed to initiate flavinylation?

The important role of a dicarboxylate molecule for covalent bond formation in complex II flavoproteins had been suggested long before it was confirmed by the biochemical studies (48). Our data (Fig. 5) demonstrate that the presence of the dicarboxylate is a prerequisite for initiation of the quinone-methide chemistry in the bacterial and mammalian systems regardless of the presence of the SdhE/SDHAF2 assembly factor. The three structures representing the flavinylation assembly intermediates provide a snapshot of the postflavinylation complexes, and they also show that the dicarboxylate ligand alters the architecture of the active site (27, 29, 30). This implies that the protein or the ligand itself can define the optimal conditions for the quinone-methide reaction. All complex II flavoproteins consist of two protein domains, a larger flavin-binding domain and a smaller capping domain (49, 50, 51, 52, 53). The unique feature of the dicarboxylate-binding site of SDHA/SdhA/FrdA is that it is located between the domains. The interdomain orientation therefore affects the binding, with interdomain motion potentially able to adjust the orientation of the substrate at the active site. Binding of malonate or oxaloacetate affects the capping domain position, specifically the position of a key catalytic Arg residue (SDHA^R340^/SdhA^R286^/FrdA^R287^) that is needed for protonation/deprotonation of the substrate during succinate and fumarate interconversion by the assembled enzymes (54, 55). In the flavinylation assembly intermediate structures, this Arg residue adopts a conformation where it is now involved in dicarboxylate binding, and in so doing places the carboxyl group(s) of the substrates within 4 Å of the isoalloxazine ring N(5)/O(4) locus (Fig. 7*B*). The change in position of the capping domain, however, does not seem to perturb the binding and interaction pattern between the protein and the isoalloxazine ring including the Arg residue (SDHA^R451^/SdhA^R399^/FrdA^R390^) required for stabilization of the imino-quinone methide intermediate negative charge near the N(1)/C(2) locus during the flavinylation reaction (Fig. 7*B*).

Our data (Fig. 2) also show that SdhE expands the number and types of carboxylate ligands that can induce flavinylation in bacterial complex II flavoproteins including monocarboxyl substrates and surprisingly even acetate. The X-ray crystal structure of the FrdA–SdhE assembly intermediate (29) was determined from crystals that contained two complexes in each asymmetric unit. The electron density for a ligand in one of these complexes was assigned as malonate, whereas in the second, it was assigned as acetate, which was a component of the crystallization conditions (29). Comparison of the two nonidentical complexes shows that acetate and one carboxyl group of malonate occupy similar positions near the isoalloxazine N(5)/C(4) region, and this carboxyl group can itself be an important factor affecting the quinine-methide chemistry (Fig. 7*C*). The substrate carboxyl group near the FAD is also hydrogen bonded to FrdA^R390^/SDHA^R451^, the residue postulated to stabilize the quinone-methide intermediate (Fig. 7, *B* and *C*). Thus, intricate interplay between FrdA^R390^/SDHA^R451^, isoalloxazine N(5)/O(4), and the substrate carboxyl may be at the center of the flavinylation mechanism in complex II flavoproteins. In addition, the flavinylation-promoting dicarboxylate ligand can enhance the stability of the flavoprotein complex with FAD by providing an extra set of polar bonds to the protein around the isoalloxazine ring (Fig. 7*D*).

The nature of the dicarboxylate ligand is also important for the flavinylation reaction in organisms that lack the *sdhE* gene or its homologs. For example, in *Thermus thermophilus*, the four carbon ligands, fumarate, succinate, malate, and oxaloacetate, were quite proficient in initiating flavinylation, and in *Sulfolobus tokodaii* succinate and fumarate, and to a lesser extent, malate also enabled proficient flavinylation (28). As our data show (Figs. 2 and 6, (27)), the assembly factor allows a greater repertoire of dicarboxylate ligands to initiate the flavinylation reaction on mesophiles presumably by helping to lock the flavoprotein conformation into an architecture that maximizes flavinylation. We suggest that these thermophilic bacteria have a more rigid protein backbone (56, 57) and do not need an assembly factor to enable facile flavinylation. Our data show that fumarate is the most potent dicarboxylate eliciting covalent flavinylation in the absence of the assembly factor. We suggest that the presence of the double bond in fumarate makes this dicarboxylate a more rigid molecule that acts as a better scaffold to restrict the active-site mobility of flavoproteins. Thus, like in thermophiles, the restricted mobility in turn enables the environment to optimize the quinone-methide chemistry needed for flavinylation.

### Why did complex II maturation evolve an assembly factor for covalent FAD?

It seems underappreciated that it is the formation of the covalent flavin bond that allows complex II to oxidize succinate (13). As oxygen arose in the atmosphere some 2.4 billion years ago (58), it might be speculated that fumarate reductases present in early forms of life acquired the ability to oxidize succinate after covalent flavin bond formation (4). In general, assembly factors may play several roles during a course of building a complex protein machinery. They are involved in maturation of individual protein subunits and/or building and stabilizing subassembly modules and coordination of final complex assembly (1, 2, 45). In case of SdhE, we hypothesize that the synchronized maturation of SDHA with SDHB, or a need for protection of an apoform of the protein, may have been an initial trigger for a new function. Cells are unusually sensitive to iron homeostasis, and accumulation of the SdhB/FrdB iron–sulfur protein may be a waste of resources and/or of potential harm from reactive oxygen species production, should maturation of SdhA but not SdhB being rate limiting. Indeed, the subassembly of complex II containing SDHA and SDHAF2 can be observed in cancer cells (35). Moreover, when complex II assembly is stalled by decreased expression of the SDHC or SDHD subunits, significant accumulation of the SDHA–SDHAF2 complex is observed while SDHB is diminished.

A comparison of the flavinylation process in SdhA and FrdA from different species shows that the decrease in efficiency of autocatalytic flavinylation correlates with the increased role of the flavinylation factor for maturation of an individual flavoprotein and assembly of the membrane-bound complex. For example, thermophilic Archaea lack an SdhE homolog, and covalent flavinylation is autocatalytic (28). In *E. coli*, deletion of *sdhE* results in a significant decrease in covalent FAD incorporation into the SdhA or FrdA proteins (32, 33); however, assembly of the intact SdhABCD or FrdABCD complexes with noncovalent FAD seems to be minimally affected (13, 34). The autocatalytic covalent FAD incorporation into SdhA/FrdA still occurs and is even boosted under microaerophilic or anaerobic growth presumably because of increased levels of fumarate (34). In mitochondria, deletion or downregulation of SDHAF2 dramatically decreases not only incorporation of covalent flavin into SDHA but also the assembly of membrane-bound complex II (41). Importantly, the critical role of the assembly factor in mitochondria directly correlates with the increased stability of the assembly complexes (27) as result of an increase in protein size due to N- and C-terminal extensions. This suggests that in addition to promoting the flavinylation reaction in SDHA, SDHAF2 has become involved in subsequent steps of the assembly of the dehydrogenase SDHAB module of complex II.

## Experimental procedures

### Plasmids and cells

*E. coli* flavoproteins SdhA and FrdA were expressed from pCA24N-SdhA and pCA24N-FrdA plasmids (59). SdhE was expressed from pQE-SdhE (29), and human apo-SDHA and SDHAF2 were both expressed from pQE-hSDHA and pST50Trc1-SDHAF2 (27). *E. coli* RP437 Δ*sdhE* (59) was used for expression of apo-SdhA and apo-FrdA, and *E. coli* BL21(DE3) was used for expression of other proteins used in this study.

### Protein expression and isolation

Apoforms of *E. coli* with an N-terminal hexahistidine tag (His_6_-SdhA and His_6_-FrdA) were expressed in SdhE KO strain RP-E as previously described (59). pCA24N-SdhA/RP-E or pCA24N-FrdA/RP-E cells were grown aerobically in LB medium supplemented with 0.1 mg/ml ampicillin at 25 °C. Protein expression was induced with 0.1 mM IPTG for 17 h at 20 ^°^C. Higher temperatures during IPTG induction resulted in increased levels of background covalent FAD incorporation probably from elevated endogenous fumarate levels in the cells. Following induction, cells were harvested by centrifugation, and the pellets were cooled on ice and resuspended in ice-cold 50 mM potassium phosphate (pH 7.5) supplemented with EDTA-free protease inhibitors (Roche) and 25 mM benzamidine. (Benzamidine is included to protect apoflavoproteins from proteolysis by OmpT protease in RP-E). To minimize proteolysis of the apoproteins, only freshly harvested cells were used for protein purification. The cell suspension was briefly sonicated and centrifuged for 40 min at ∼100,000*g*. The obtained cell lysate was loaded onto a nickel–nitrilotriacetic acid column and washed with 50 mM potassium phosphate (pH 7.5), 30 mM imidazole, 0.3 mM NaCl, and 5% glycerol. The protein was eluted with 0.25 M imidazole followed by concentration and buffer exchange with 20 mM Hepes (pH 7.5) and 10% glycerol using centrifugal concentrators (Millipore; 50 K). Expression and purification of SdhE followed the aforementioned protocol, except Millipore 10 K concentration units were used. Purified proteins were aliquoted and stored at −80 °C until use. Human apo-SDHA and SDHAF2 were modified with an N-terminal His_6_ tag and purified as previously described (27).

### *In vitro* flavinylation reaction

Unless specified, the reaction was performed in 50 mM potassium phosphate (pH 7.0) at 30 ^°^C for 1 h. Apoflavoproteins (2.8 μM, or as indicated) were mixed with 100 μM FAD and the corresponding assembly factor, that is, SdhE for the bacterial proteins (10.4 μM) and SDHAF2 for human SDHA (7.2 μM). The reaction was started after addition of the appropriate flavinylation-promoting ligand at the as-indicated concentration. At different time points, the samples were mixed with 4× SDS loading buffer (Bio-Rad), and the sample loaded onto an SDS-PAGE gel (Bio-Rad; Any KdA). Following protein separation, the gel was incubated in 10% acetic acid/20% ethanol for 5 min. Upon illumination with UV light, complex II flavinylated flavoprotein is visible because of the fluorescence of the covalently bound flavin. The in-gel fluorescence of covalently attached FAD was detected using a Safe Imager 2.0 blue-light transilluminator (Thermo Fisher Scientific). The gel was then rinsed with water for 5 min and stained with Instant Blue stain (Expedeon). ImageJ (National Institutes of Health) software was used for quantification of covalently bound FAD levels and relative protein ratios. The isolated holo-SdhA/FrdA or SDHA–SDHAF2 complex were used as 100% covalent flavinylated controls. The flavinylation reaction of SdhA in the presence of SdhE and fumarate was rapid requiring a faster time point collection. To do that, upon addition of fumarate, the sample (200 μl) was quickly mixed and filled into an automatic pipette. Each sample (15 μl) was then injected into a vial containing 5 μl of 4× SDS loading gel. This allowed collection of time points that are at 10 to 15 s intervals.

Spectral measurements and kinetics were performed by using an Agilent 8435 UV–visible spectrophotometer. For kinetic measurements, whole spectra (1 nm increments) were collected during the reaction at 1 to 5 s intervals. Reduction/oxidation 0of FAD was monitored in a 0.2 ml optical cuvette containing 50 mM potassium phosphate (pH 7.5), 25 μM apo-SdhA, 20 μM FAD, and 40 μM SdhE at 30 ^°^C following the addition of 10 mM fumarate. Anaerobic conditions were achieved in the presence of 10 mM glucose, 20 μg/ml glucose oxidase, and 5 μg/ml catalase with an argon-filled head space.

### Determination of *K*_*d*_ for FAD by ultrafiltration

The method is adapted from Mortarino *et al.* (36). Apo-SdhA (2.8 μM) in 0.5 ml of 50 mM Hepes, pH 7.0, containing 8 μM FAD with or without 12.5 μM SdhE was incubated at 21 ^°^C for 20 min and concentrated to ∼250 μl by centrifugation (1 min at 15,000*g*) using 30 K molecular weight cutoff 0.5 ml protein concentrators from Pierce. The exact volume and the concentration of free flavin (*ε*^*450*^ = 11.3 mM^−1^ cm^−1^) in the filtrate was measured, and it is assumed the free flavin (FAD^*free*^) concentrations are the same in the filtrate and concentrated fractions. The total FAD in the concentrated fraction was calculated, and the excess over FAD^*free*^ corresponded to SdhA–noncovalent FAD. The *K*_*d*_ = [FAD^*free*^][apo-SdhA]/[SdhA–FAD] was calculated from the amount of apo-SdhA determined by total protein concentration. The data are expressed as mean ± SD of four to five independent measurements.

### Formation of hydrogen peroxide upon covalent flavinylation of SdhA

Upon covalent attachment, the flavin in SdhA became reduced and can be reoxidized by oxygen with formation of hydrogen peroxide. Amplex UltraRed was used to monitor the hydrogen peroxide formed. The reaction was performed in 50 mM potassium phosphate (pH 7.0) at 25 ^°^C in the presence of 2.8 μM apo-SdhA, 4.8 μM SdhE, 2.5 μM FAD, 20 μM Amplex UltraRed, and hydrogen peroxidase. The reaction was started by addition of either 10 μM or 10 mM fumarate and hydrogen peroxide monitored by following the oxidation of Amplex UltraRed (567 nm) using an Agilent 8453 spectrophotometer.

### Testing stability of *E. coli* apo-SdhA and human apo-human SDHA

Apo-SdhA (2.8 μM) or apo-SDHA (4.3 μM) in 40 mM Hepes (pH 7.5) was incubated at 37 ^°^C as follows: (1) no addition; (2) FAD (100 μM); (3) the assembly factors SdhE (10.4 μM) or SDHAF2 (8.4 μM); and (4) FAD (100 μM) plus appropriate assembly factors at the aforementioned concentrations. The incubation time was 15 min for SDHA and 30 min for SdhA. Corresponding control samples (with no additions) were held at 4 ^°^C for the same time. After the incubation, the samples were transferred to 4 ^°^C, and to all samples, including the control, either FAD and/or the assembly factor was added so that each sample contains 100 μM FAD and the appropriate assembly factor. Then 0.5 mM fumarate is added to each sample to initiate the flavinylation reaction. After 30 min incubation at 30 ^°^C, 4× SDS sample buffer was added, and then in-gel fluorescence was determined as described previously.

### Analytical methods

Protein concentration was determined by the bicinchoninic acid protein assay kit from Pierce.

## Data availability

Data will be made available upon reasonable request, made to Gary Cecchini, Gary.Cecchini@ucsf.edu.

## Conflict of interest

The authors declare that they have no conflicts of interest with the contents of this article.
